# Simulation of Large Scale Neural Models With Event-Driven Connectivity Generation

**DOI:** 10.3389/fninf.2020.522000

**Published:** 2020-10-14

**Authors:** Nathalie Azevedo Carvalho, Sylvain Contassot-Vivier, Laure Buhry, Dominique Martinez

**Affiliations:** ^1^Université de Lorraine, CNRS, Inria, LORIA, Nancy, France; ^2^Université de Lorraine, CNRS, LORIA, Nancy, France

**Keywords:** brain simulation, Hodgkin-Huxley neurons, time-stepping method, event-driven connectivity generation, Runge-Kutta method, Parkinson's disease, large scale networks

## Abstract

Accurate simulations of brain structures is a major problem in neuroscience. Many works are dedicated to design better models or to develop more efficient simulation schemes. In this paper, we propose a hybrid simulation scheme that combines time-stepping second-order integration of Hodgkin-Huxley (HH) type neurons with event-driven updating of the synaptic currents. As the HH model is a continuous model, there is no explicit spike events. Thus, in order to preserve the accuracy of the integration method, a spike detection algorithm is developed that accurately determines spike times. This approach allows us to regenerate the outgoing connections at each event, thereby avoiding the storage of the connectivity. Consequently, memory consumption is significantly reduced while preserving execution time and accuracy of the simulations, especially the spike times of detailed point neuron models. The efficiency of the method, implemented in the **SiReNe** software^1^, is demonstrated by the simulation of a striatum model which consists of more than 10^6^ neurons and 10^8^ synapses (each neuron has a fan-out of 504 post-synaptic neurons), under normal and Parkinson's conditions.

## 1. Introduction

**Figure 1:**
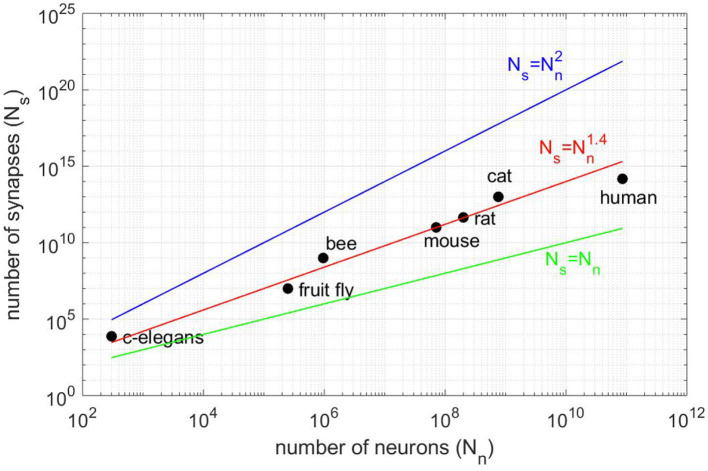
Number of synapses *N*_*s*_ vs. number of neurons *N*_*n*_ for different animal species (vertebrates and invertebrates). The best fit is for the power law $N_{s}=N_{n}^{1.4}$ (red line) and not for *N*_*s*_ = *N*_*n*_ (black line), as claimed in Lansner and Diesmann (2012), or the full connectivity $N_{s}=N_{n}^{2}$ (blue line). All fits are consistent in the sense that *N*_*s*_ = 0 for *N*_*n*_ = 0.

Major projects such as Blue Brain (Markram, 2006), the Human Brain Project (Einevoll et al., 2019), the BRAIN Initiative or Mindscope (Hawrylycz et al., 2016), aim at simulating a brain or brain structures. The simulation of brain structures demands not only computing resources but also a lot of memory to store the connectivity that grows as the power 1.4 of the number of neurons (see Figure 1). Then, the simulation of the human brain would require ≈100 terabytes just for storing the Boolean connectivity pattern (connection/no connection). Therefore, large-scale simulations of realistic cortical networks have been undertaken by using supercomputers with huge memory space (Izhikevich and Edelman, 2008; Migliore et al., 2006; Chatzikonstantis et al., 2019; Eliasmith and Trujillo, 2014).

Here, we propose a hybrid approach that combines a time-stepping approach for the numerical part of the simulation with an event-driven updating of the synaptic currents for complex Hodgkin-Huxley (HH) type neurons. This approach is particularly well-suited to studies of real-time neural mechanisms requiring high accuracy, such as pathological behaviors like the Parkinson's disease. Our event-driven approach completely avoids the storage of the connectivity pattern by regenerating the connectivity on the fly, when needed, after spiking events. This event-driven generation of the connectivity makes use of pseudo-random generators and consistent seeds. The exact computation of spike times is not possible for HH neurons because the model is continuous and the membrane voltage is approximated by time-stepping methods on discrete time (Koch and Segev, 1998). Moreover, when the membrane potential crosses threshold twice during one time-step (the first crossing is upward and the second one is downward), the spike may be missed. A failure to detect a spiking event may cause dramatic changes on the behavior of the system, especially in the case of event-driven connectivity.

In this paper, we consider an event-driven connectivity generation within time-stepping schemes of Runge-Kutta 2 midpoint type. We develop a spike detection method for HH neurons, that accurately determines the spike timings so that the accuracy of the second-order Runge-Kutta methods (RK2) is preserved when connectivity is generated at spiking events. By avoiding the connectivity storage, our method is intended to simulate large-scale models made of Hodgkin-Huxley type neurons on a single computing node. Indeed, the limited memory consumption pushes back the necessity to use multiple machines, whose induced communications often reduce the overall performance. Yet, computing performance is not neglected in our approach as a parallel multi-threaded version has been developed in order to take advantage of multi-core/many-core machines.

Our approach is implemented in the **SiReNe** software whose accuracy and efficiency are exhibited in a series of experiments among which is the simulation of the striatum structure at the rat scale, i.e., with more than 10^6^ neurons.

In the next section, we demonstrate the originality and interest of our approach in respect to related works. The different methods used in our neural simulator are presented in section 3. Then, validation and performance analysis are provided in section 4. Section 5 draws a general conclusion and proposes a list of future works.

## 2. Related Works

### 2.1. Event-Driven Connectivity Generation

In neuroscience studies, one often simulates a snapshot of a network over a short period of time with fixed parameters, e.g., for comparing normal vs. pathological neural configurations. In such a context, the causal mechanisms (e.g., plasticity mechanisms) of neural evolution from normal to pathological states is not relevant. Thereby, connectivity storage is not mandatory and can be advantageously replaced by dynamic generation. Originally, the idea of event-driven connectivity generation has been proposed in the case of abstract neurons for which spike timing is exactly known, i.e., rule-based artificial cell units, or finite state machines (Lytton and Stewart, 2006). This approach has then been applied with integrate-and-fire (IF) neurons, i.e., quadratic IF (Izhikevich and Edelman, 2008) and leaky IF over GPU hardware (Knight and Nowotny, 2020).

To the best of our knowledge, the event-driven connectivity generation approach has never been developed for Hodgkin-Huxley neurons. Our event-driven connectivity generation makes use of pseudo-random generators and consistent seeds. The principle of pseudo-randomly generating the connectivity through an event-driven approach has been reused in a recent US patent (Lipasti et al., 2019). Yet, the LFSR (Linear-Feedback Shift Register) nature of the pseudo-random number generator (PRNG) used in their approach is not statistically strong as it does not pass classical linear tests provided in TestU01 (L'Ecuyer and Simard, 2007) which is a reference in the domain. Moreover, a single PRNG is used to perform all the dynamic data generations, leading to additional time and memory consumption.

### 2.2. Spike Detection

Traditionally, event-driven strategies are applied at the neuron level (Makino, 2003; Brette, 2006, 2007; Tonnelier et al., 2007; Rochel and Martinez, 2003; Mattia and Del Giudice, 2000; Ros et al., 2006; Morrison et al., 2007; Rhodes et al., 2018). In pure event-driven strategies the spike timings are analytically given and are calculated with an arbitrary precision (up to the machine precision). This scheme allows for an exact simulation where no spike is missed. Yet, only a limited class of simplified neuron models of integrate-and-fire (IF) type is amenable to exact simulations. Thus, more complex neuron models are simulated on discrete times by using time-stepping methods, e.g., the second order RK2 algorithm. Nevertheless, aligning the spike times on the time grid leads to an accuracy of order one, as for example in Lipasti et al. (2019). For IF neurons, the determination of the spike times by linear interpolation is needed to preserve the order 2 of the RK2 method (Hansel et al., 1998; Shelley and Tao, 2001). In contrast to IF neurons, HH neurons do not have an explicit threshold so that determining the spike times by threshold crossing, as in Lobb et al. (2005), or by linear interpolation, as for IF neurons, will lead to low accuracy. Here, we show that for HH neurons, a quadratic interpolation (e.g., Bézier curves) is required to be consistent with the order 2 of the RK2 method. In contrast to Morrison et al. (2007) in which quadratic and cubic interpolations are considered, it is not necessary to use more than a quadratic interpolation. Actually, cubic (and higher-order) interpolation should be avoided as it implies additional computation cost for no gain in accuracy (Hansel et al., 1998).

### 2.3. Parallel Computation

Many studies have been done about the use of parallelism in neural simulations (Kunkel et al., 2012; Lansner and Diesmann, 2012; Kunkel et al., 2014; Jordan et al., 2018). Yet, most of them use connectivity storage, implying a huge memory consumption and the resort to distributed parallelism. By using event-driven connectivity generation, the memory requirements are significantly reduced and it becomes possible to simulate very large networks on a single computing node. In such a context, distributed parallelism is interesting merely for the increased computational power it offers, as compared to a single many-core node. However, it is worth noting that distributed parallelism implies additional overhead due to data communications between machines, which may significantly reduce the interest of the distribution. Consequently, we propose in this paper a multi-threaded parallel version of event-driven updating and connectivity generation that can run efficiently on a single multi-core node.

## 3. Methods

In the following sections, we present the different types of simulation schemes: the common time-stepping approach, the event-driven connectivity generation, as well as the spike detection method we developed. Then, we describe the striatum model and use it to test the different methods. Finally, we present the implementation in the **SiReNe** software.

### 3.1. General Simulation: Time-Stepping

**Figure 2:**
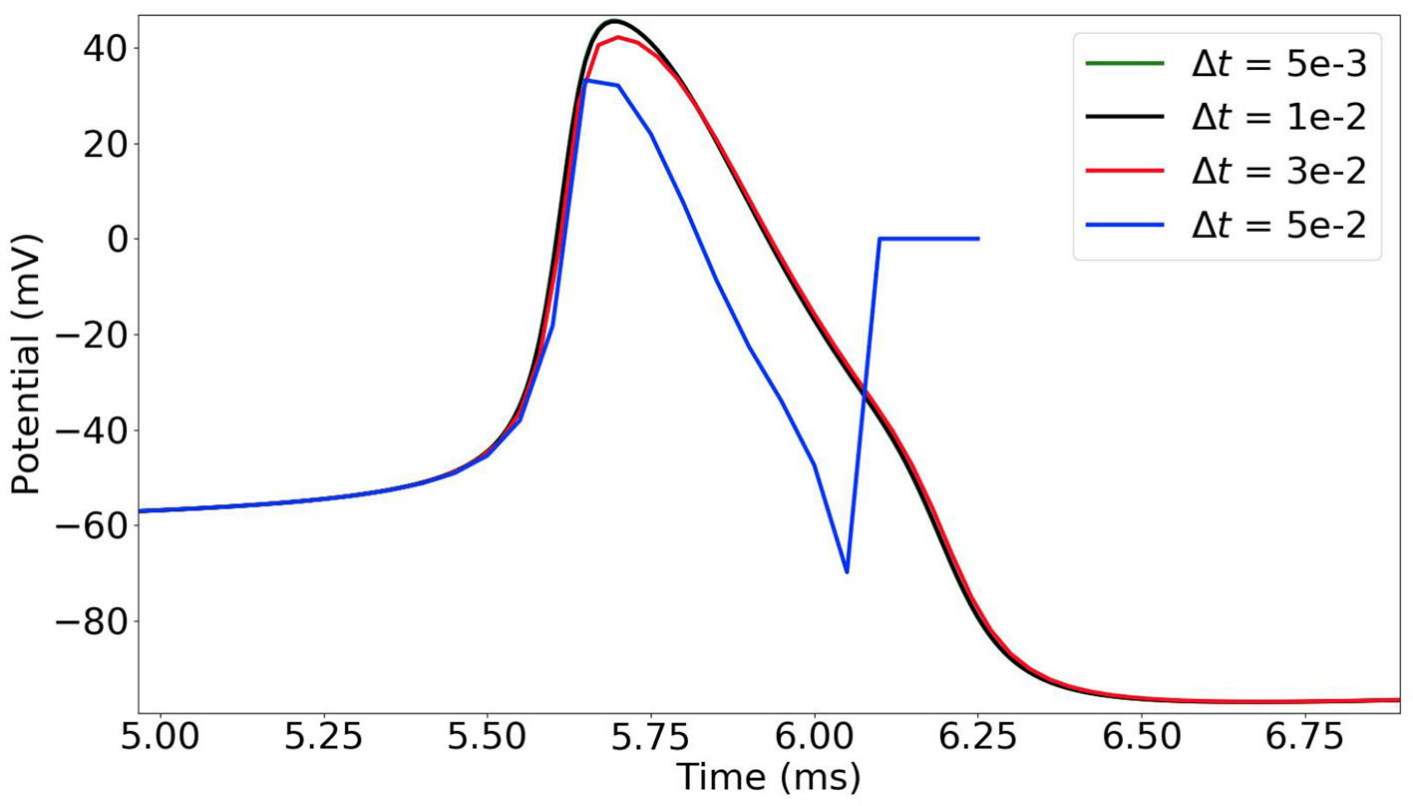
Time-stepping simulation of a single MSN neuron with different time-steps Δ*t* = 0.05, 0.03, 0.01 and 0.005 ms (plot of the first action potential).

In the time-stepping approach, the state variables of the neurons are updated at each time-step (Δ*t* = 0.005 ms in our simulations). As the dynamics of the neurons is highly non-linear and sharply varies, the choice of the time-step must be thoroughly considered. In Figure 2, we observe that when the time-step is too large (e.g., 0.05 ms) the simulation results are incorrect. With a time-step of 0.03 ms, the potential curve begins to take the right shape, and it gets more accurate with smaller time-steps.

The ordinary differential equations of the Hodgkin-Huxley neurons type (HH) are solved by an explicit iterative temporal discretization method, i.e., Runge-Kutta 2 (RK2) in our case. The RK2 method is a second-order method leading to an error *O* (Δ*t*^2^) of order two.

In classical time-stepping simulations, the synaptic current of each neuron is updated at each time-step. The pre-synaptic neurons are used at each step (Lytton et al., 2008) to perform the updating of the synaptic current. However, this approach is very time consuming and, most of the time, partly useless, as only spiking neurons generate post-synaptic currents. We propose in the next section a hybrid simulation scheme with event-driven post-synaptic updating that avoids useless computations.

### 3.2. Event-Driven Connectivity Generation and Post-synaptic Updating

In the hybrid simulation scheme that we propose, an event-driven strategy is applied at the connectivity level. The idea is to be more pro-active by changing the order of the synaptic updating process. Instead of computing the state of the synaptic currents at time *t* according to previous time *t* − Δ*t*, the updating scheme executes the steps given in Algorithm 1.

**Algorithm 1:**
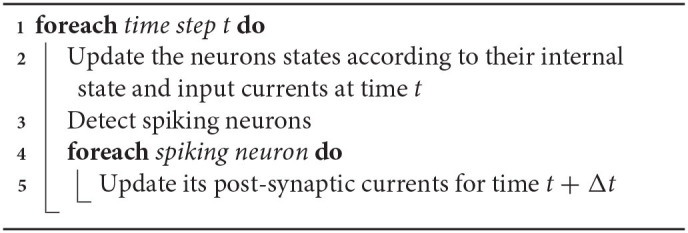
Simulation scheme

The advantage of this method is to update the post-synaptic neurons of *only* the firing neurons, whereas in classical time-stepping simulations, pre-synaptic neurons of *all* neurons are systematically updated, even those that have not fired recently. So, this event-driven updating scheme can significantly reduce the overall computations when only a fraction of the neurons fires in the same time-step. Moreover, this method can be combined to a pseudo-random generation of the connectivity, that significantly reduces the memory cost of the simulation.

#### 3.2.1. Event-Driven Generation of the Connectivity

In order to avoid the storage of the entire connectivity of the neural system, and thereby to limit the memory consumption, the set of post-synaptic neurons as well as the intrinsic synaptic parameters (peak conductance,...) can be pseudo-randomly generated after each spike (Lytton et al., 2008). Synaptic parameters are defined for each synaptic model, and individuation can be obtained either by a specific computation based on the neuron number or by the addition of noise.

The pseudo-random nature of the generation ensures that the generated sequence is reproducible for a same seed. Thus, the connectivity of a neuron stays the same during the entire simulation as long as the same seed is used for that neuron. Also, the use of distinct seeds and internal PRNG states between the neurons implies that the connectivity is different from a neuron to one another. Consequently, when a neuron fires, its post-synaptic connections are pseudo-randomly generated according to its corresponding random seed and internal PRNG state, and the synaptic current of each post-synaptic neuron is updated.

In this context, the number of post-synaptic neurons of every neuron is fixed at the simulation initialization. This number can be provided by the user, either as an absolute value or through a connection density. Then, there is a need for an algorithm that uniformly draws *M* elements (post-synaptic neurons) from the integers set [1, *N*] (all possible candidates) without repetition. One practical solution is to sequentially parse the set of candidates and to perform a pseudo-random selection according to a probability defined in function of the number *r*_*c*_ of remaining candidates and the number *r*_*s*_ of elements still to be selected. Therefore, when considering candidate neuron *i*, the probability to select *i* is given by $P\left(i\right)=\frac{r_{s}}{r_{c}}$. It can be checked that, by construction, this value is defined in [0, 1]. Also, the probability distribution of the selected connections obtained by this process is definitely uniform. In fact, for drawing the *M* elements, other solutions are possible that are theoretically more accurate in terms of probabilities. Indeed, the smallest value of *k* reals randomly drawn in the interval [*x*, 1] (0 ≤ *x* ≤ 1) is theoretically given by 1 − (1 − *R*)^1/*k*^ × (1 − *x*), where *R* is a random real number in [0, 1]. Then, an additional step is required to obtain integer values. Obviously, such a method is much more computationally expensive than the one we use. Indeed, our choice is a good compromise between computation efficiency and distribution quality of the candidates selection.

In its current form, our simulator uses different types of neurons, such as excitatory vs. inhibitory neurons within the same neural group or distinct neurons belonging to different groups. A type of synapse is thus defined (with peak conductance and time constant parameters) between two types of neurons (source type and destination type). Consequently, the memory storage devoted to the synapses scales at most as the square of the number of neuron types and not as the square of the number of neurons. Also, a neuron in one group may be connected to any neurons in its own group and in the other groups. Its post-synaptic connections are generated by selecting for each post-synaptic group of the neuron, the number of post-synaptic neurons defined by the connection density between the neuron group and that particular post-synaptic group. Then, the parsing and pseudo-random selection process described above is applied independently to each post-synaptic group, using the pseudo-random draw presented in Algorithm 2.

**Algorithm 2:**
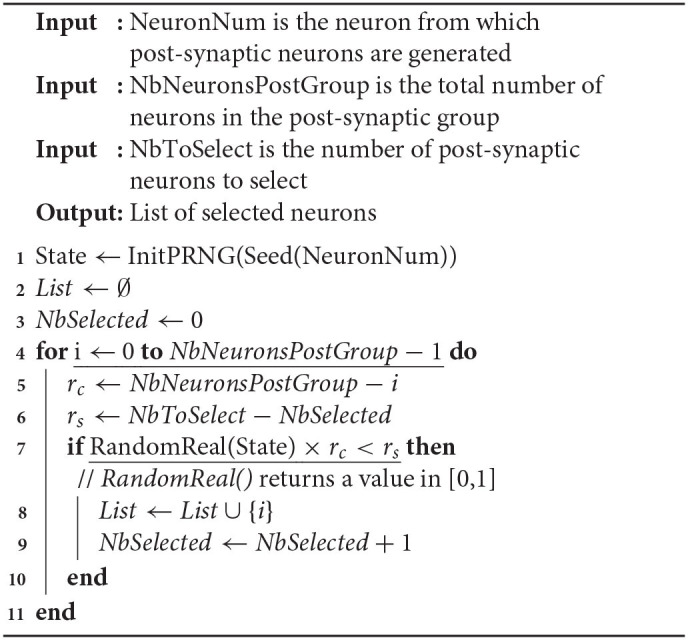
Pseudo-random connectivity generation

In this algorithm, the function Seed() generates a unique seed from the neuron number. Although this is not mandatory, it may be designed to ensure a minimal distance between seeds of neurons with similar connectivity parameters, in order to avoid similarities in the distances between selected post-synaptic neurons. The function InitPRNG() initializes the internal state of the PRNG so that distinct states are produced from distinct seeds. Then, the internal state is used and updated in function RandomReal() that draws a random value in [0, 1]. Also, the list of selected post-synaptic neurons is not stored in memory in practice, but the corresponding synaptic currents are directly updated on the fly. When the post-synaptic group is the same as the neuron group, the neuron NeuronNum can be excluded from the list of post-synaptic neurons in order to avoid autapses. In such a context, the loop in line 4 must be divided in two loops, according to the neuron indices that are lower or greater than NeuronNum.

Finally, in order to get a good distribution quality of the PRNG while preserving the performance constraint (generation speed), a fast and robust generator must be chosen. In our simulator, the Lehmer64 generator is used (derived from Lehmer, 1951), as it is one of the fastest generator that passes the Big Crush of TestU01 (L'Ecuyer and Simard, 2007), a battery of statistical tests that is a reference for quality evaluation of PRNGs. Moreover, as this PRNG works on 64 bits, it provides 2^64^ ≈ 10^19^ seeds, which is much more than the number of neurons in the human brain (≈ 10^11^).

#### 3.2.2. Event-Driven Computation of Synaptic Currents

Time evolution of the synaptic conductance *g*_*jk*_ between pre-synaptic neuron *j* and post-synaptic neuron *k* is modeled by the following differential equation

(1) $$\begin{array}{l} \frac{dg_{jk}}{dt}=-\frac{g_{jk}}{\tau_{jk}} \end{array}$$

with τ_*jk*_, the time constant of the synapse. Integrating the equation between *t* (current time) and *t*_*sp*_ (spike time) leads to

(2) $$\begin{array}{l} g_{jk}\left(t\right)={ḡ}_{jk}\exp\left(-\frac{t-t_{sp}}{\tau_{jk}}\right) \end{array}$$

with ḡ_*jk*_ = *g*_*jk*_(*t*_*sp*_), the peak conductance. The total synaptic current *I*_*syn,k*_ for neuron *k* is the sum of the contributions of the pre-synaptic neurons *j* and all their pre-synaptic spikes

(3) $$\begin{array}{l} I_{syn,k}\left(t\right)=\underset{j}{\sum }g_{jk}\left(t\right)\left(V_{k}-E_{jk}\right)\\ \;=\underset{j}{\sum }{ḡ}_{jk}\left(V_{k}-E_{jk}\right)\underset{Fact_{jk}\left(t_{sp_{n_{j}}}\right)}{\underset{︸}{\overset{n_{j}}{\underset{i=1}{\sum }}\exp\left(-\frac{t-t_{sp_{i}}}{\tau_{jk}}\right)}} \end{array}$$

where *E*_*jk*_ is the reversal potential of the synapse from neuron *j* to *k* and *V*_*k*_ is the membrane potential of the neuron *k*. The number of spikes received from neuron *j* is denoted *n*_*j*_ and *t*_*sp*_*i*__ represents the firing time of the *i*^*th*^ spike. *Fact*_*jk*_ is updated at each event (i.e., when neuron *j* emits a spike) as follows

(4) $$\begin{array}{l} \;n_{j}\leftarrow n_{j}+1\\ Fact_{jk}\leftarrow Fact_{jk}+\exp\left(-\frac{t-t_{sp_{n_{j}+1}}}{\tau_{jk}}\right) \end{array}$$

### 3.3. Event Detection

Spikes are detected when the membrane potential exceeds a given threshold. In classical methods, the firing time is then aligned to the time-step. Yet, this trivial detection method leads to an error *O* (Δ*t*) that is not consistent with the RK2 method (Hansel et al., 1998). From a numerical point of view, the firing time should be at the maximum of the membrane potential. Herein, we propose two interpolation methods to obtain a more accurate estimation of the firing time. The former is based on the intersection between two linear interpolations and the latter is based on a Bézier curve. As shown in section 4, only the latter leads to an error *O* (Δ*t*^2^) that is consistent with the RK2 method.

#### 3.3.1. Intersection Between Linear Interpolations

**Figure 3:**
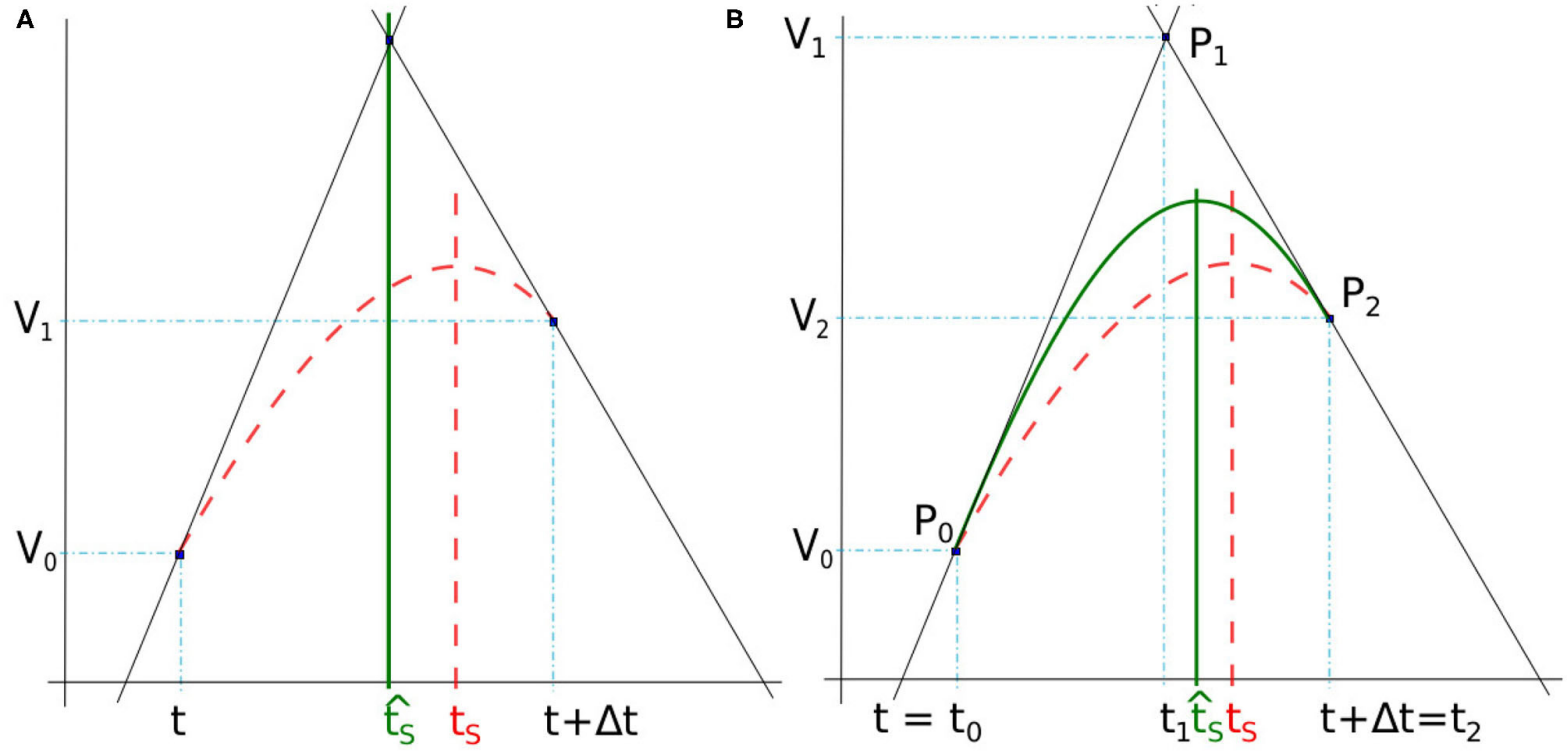
**(A)** Spike time ${\hat{t}}_{S}$ obtained by lines intersection issued from derivatives at *t* and *t* + Δ*t* (full green line). **(B)** Spike time ${\hat{t}}_{S}$ detected with the maximum of a quadratic Bézier curve (full green line) defined between *t* = *t*_0_ and *t* + Δ*t* = *t*_2_. In both figures, the reference time spike *t*_*S*_ is obtained with 10^−7^ simulation time-step (dashed red line).

Here, the spike time *t*_*S*_ is found at the intersection between two lines deduced from the membrane potential derivatives at the time-step frontiers (Figure 3A). In our case, the slopes of the two lines are defined by the derivatives *dV*_0_/*dt* and *dV*_1_/*dt* where *V*_0_ and *V*_1_ are the membrane potential at *t* and *t* + Δ*t*, respectively. Note that when the time-step is small enough, the top value of the spike takes place inside a time-step whose derivatives at start and end times (*t* and *t* + Δ*t*) are positive and negative, respectively.

If we define $b_{0}=V_{0}-\frac{dV_{0}}{dt}t$ and $b_{1}=V_{1}-\frac{dV_{1}}{dt}\left(t+\Delta t\right)$, the equation of the two lines can be written as

(5) $$\begin{array}{l} y=\frac{dV_{i}}{dt}x+b_{i}\text{ with }i\in \left\{0,1\right\},\;\forall x\in \left[t,t+\Delta t\right] \end{array}$$

where *y* ∈ ℝ. The spike time estimate ${\hat{t}}_{S}$ found at the intersection of the two lines is given by

(6) $$\begin{array}{l} {\hat{t}}_{S}=\frac{b_{1}-b_{0}}{\frac{dV_{0}}{dt}-\frac{dV_{1}}{dt}} \end{array}$$

Although this method provides good results in some cases, it has not been selected for use in our simulator as it is not accurate enough in general. As a consequence, we have chosen a method of higher order that makes a more meaningful use of the three points given above (intersection and the two time-step boundaries), by interpreting the two derivatives at *t* and *t* + Δ*t* as the tangents of a quadratic Bézier curve.

#### 3.3.2. Bézier Curve

The Bézier curve of order two is a polynomial curve specified by three points *P*_0_, *P*_1_, and *P*_2_ that define the tangents *P*_0_*P*_1_ and *P*_1_*P*_2_, respectively at starting point *P*_0_ and ending point *P*_2_. Its parametric form *B*(*x*) with *x* ∈ [0, 1] is given by

(7) $$\begin{array}{l} B\left(x\right)={\left(1-x\right)}^{2}\;P_{0}+2\left(1-x\right)\;xP_{1}+x^{2}P_{2} \end{array}$$

As illustrated in Figure 3B, the spike time ${\hat{t}}_{S}$ is estimated from the interpolation of the membrane potential with a quadratic Bézier curve, the points *P*_*i*_ = (*t*_*i*_, *V*_*i*_), with *i* ∈ {0, 1, 2}, are defined at times *t*_*i*_ and potentials *V*_*i*_. The end points of the curve, *P*_0_ and *P*_2_, are given by the potentials *V*_0_ and *V*_2_ at times *t*_0_ = *t* and *t*_2_ = *t* + Δ*t*. The point *P*_1_ is defined as the intersection of the two lines following the derivatives in *P*_0_ and *P*_1_, similarly to section 3.3.1. The time *t*_1_ is determined as ${\hat{t}}_{S}$ in Equation (6), and *V*_1_ is deduced from Equation (5) and (6), as

(8) $$\begin{array}{l} V_{1}=\frac{b_{2}\frac{dV_{0}}{dt}-b_{0}\frac{dV_{2}}{dt}}{\frac{dV_{0}}{dt}-\frac{dV_{2}}{dt}} \end{array}$$

Once *P*_0_, *P*_1_, and *P*_2_ are obtained, ${\hat{t}}_{S}$ is analytically computed as the time at which the Bézier curve reaches a null derivative (curve peak), i.e.,

(9) $$\begin{array}{l} {\hat{t}}_{S}={\left(1-\hat{x}\right)}^{2}\;t_{0}+2\hat{x}\;\left(1-\hat{x}\right)\;t_{1}+{\hat{x}}^{2}t_{2} \end{array}$$

where $\hat{x}\in \left[0,1\right]$ is defined such that $\frac{dB}{dx}\left(\hat{x}\right)=0$, leading to $\hat{x}=\frac{V_{0}-V_{1}}{V_{0}-2V_{1}+V_{2}}$. The particular case where *V*_0_ − 2*V*_1_ + *V*_2_ = 0 does not occur in practice as it would mean that the spike extremum is constant over the time step. Indeed, the Bézier interpolation is performed only when the derivatives at the time step bounds are not null.

### 3.4. Simulated Striatum Model

The striatum is a brain structure presumably involved in generating pathological β-oscillations observed in Parkinson's disease (McCarthy et al., 2011; Corbit et al., 2016). The striatum is composed in its vast majority (≈ 95%) of **M**edium **S**piny **N**eurons (**MSN**) (Kemp and Powell, 1971; Corbit et al., 2016). Our MSN model equations and the ionic channel are derived from the (McCarthy et al., 2011) model.

The voltage *V* at each time-step is described by:

(10) $$\begin{array}{l} C_{m}\frac{dV}{dt}=-\underset{I_{K}}{\underset{︸}{{ḡ}_{K}n^{4}\left(V-E_{K}\right)}}-\underset{I_{Na}}{\underset{︸}{{ḡ}_{Na}m^{3}h\left(V-E_{Na}\right)}}\\ \;-\underset{I_{M}}{\underset{︸}{{ḡ}_{M}p\left(V-E_{K}\right)}}-{ḡ}_{L}\left(V-E_{L}\right)-I_{syn}+I_{app} \end{array}$$

where *C*_*m*_ is the membrane capacitance, ḡ_*X*_ is the maximal conductance of ion *X*. The values *h*, *m*, *n*, and *p* are the respective gating variables of the different ions channels (activation/inactivation). Also, *E*_*X*_ is the reversal potential of the ion channel *X*. The fast potassium current *I*_*K*_ has four activation gates and no inactivation gate. The sodium current *I*_*Na*_ has three activation gates and one inactivation gate. M-current is a non inactivating potassium current which has one activation gate and no inactivation gate. The leak current is denoted by *I*_*L*_. The synaptic current *I*_*syn*_ is a GABAa inhibitory current given in Equation (3) with *E*_*jk*_ = −80 mV and ḡ_*jk*_ is between $\frac{0.1}{N}$ mS and $\frac{0.6}{N}$ mS, where *N* is the number of synaptic connections. The applied current *I*_*app*_ is a step current, i.e., at the beginning it is −10 μ A and at *t* = 500 ms it goes up to 0.4 μ A. We also add a uniform noise between −*b* and *b* to the applied current.

Since the idea of our article is to promote the simulation of large-scale networks, we focus on the simulation of the rat's MSN network at scale one. This network is composed of about 1.3 million neurons (Oorschot, 1996). We consider that a MSN neuron is surrounded by ≈ 2800 other MSN neurons and that the connection density in this neighborhood is of 18%, as in Taverna et al. (2008) and Lindahl and Hellgren Kotaleski (2017)), i.e., the number of post-synaptic neurons per MSN would be ≈ 504 neurons.

We thus simulate a MSN model of 1.3 million neurons with 504 connections per neuron. To demonstrate the efficiency of our simulation approach, we compare our results to McCarthy et al. (2011), during a 4 s biological time simulation. In all our simulations, the time-step is fixed to Δ*t* = 0.005 ms and the Bézier's curve is used to interpolate the spike times. The **L**ocal **F**ield **P**otential (LFP) is the sum of the synaptic currents of each neuron at each time-step (McCarthy et al., 2011). The LFP power spectrum was deduced from a standard Fourier Transform Function.

Further tests are done to compare classical time-stepping and event-driven updating approach, in terms of memory consumption and execution times. In this context, we simulate 100, 500, 1000, 5000 and 10000 neurons for 100 ms biological time.

Finally, a last experiment is done to evaluate the performance of the parallel multi-threaded version with OpenMP. We compare execution times for a 1 s biological simulation time with 10000 MSN neurons as a function of the number of threads (1, 2, 4, 8, 16, and 32).

### 3.5. Software Implementation in SiReNe

**Figure 4:**
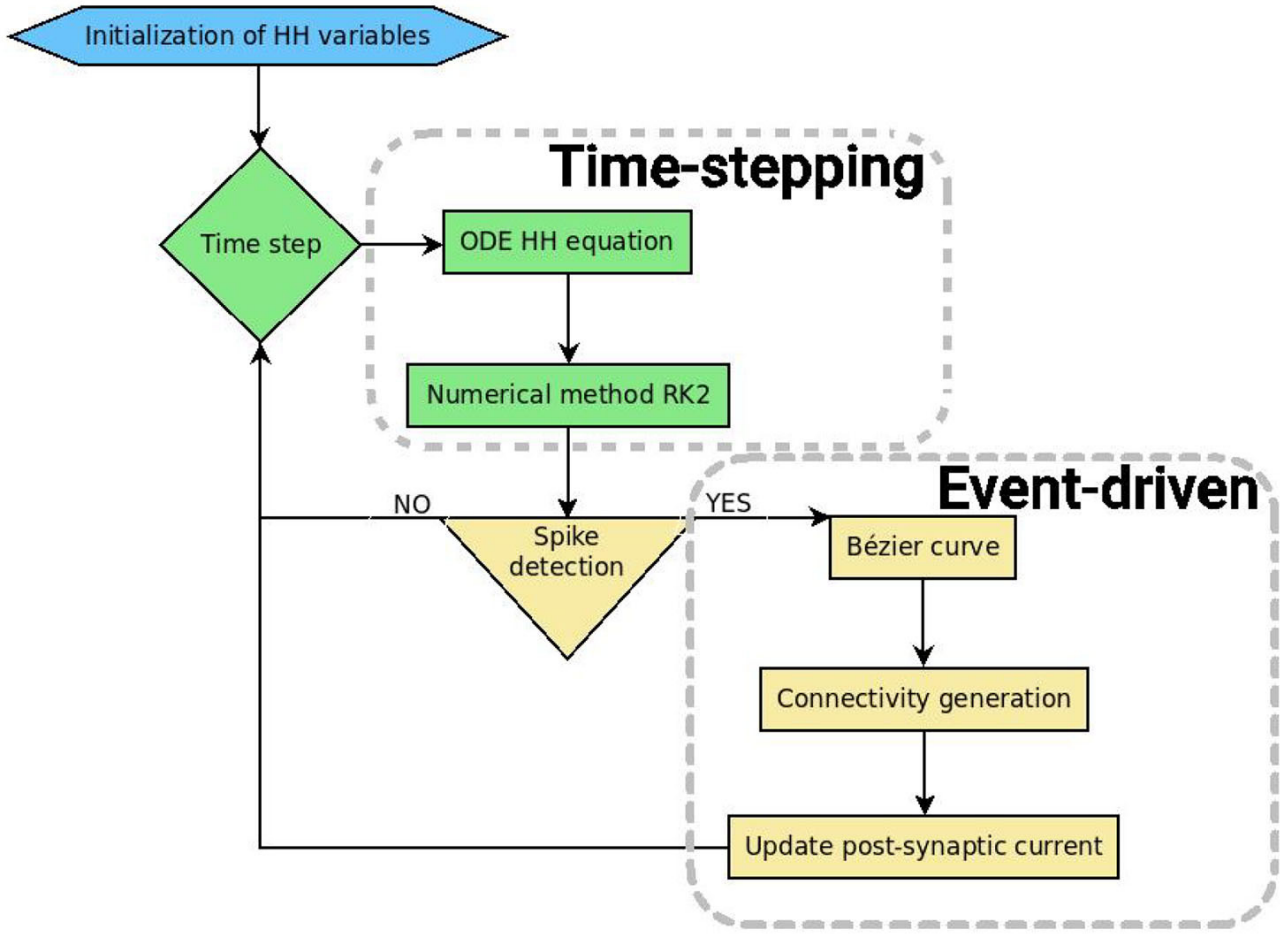
Schematic representation of the **SiReNe** software (implemented in C).

**SiReNe** (“***Si****mulateur de* ***R*é***seaux de* ***Ne****urones*,” “Neural Network Simulator” in English) software, is a C program that has been developed in our laboratory for several years. Originally, it is a pure time-stepping simulator, but it has been extended with the event-driven updating method presented in this paper. So, it is now able to simulate large neural networks either with a classical time-stepping method, or with the event-driven one. In both approaches, the non-linear differential equations of the HH model are updated with the help of the RK2 integration method. After updating all state variables, it is checked whether the neurons have fired. The potential of the neuron has to cross a threshold during this time-step and the derivatives of the potential at times *t* and *t* + Δ*t* must be respectively positive and negative. In order to detect the spikes, the time delay of each neuron has to be larger than the time-step, otherwise some spikes may be missed. In the pure time-stepping version, the pre-synaptic currents are systematically retrieved for every neuron, whereas in the event-driven version, neurons are updated first, then only the post-synaptic connections of the spiking neurons are generated and their currents are updated. Figure 4 shows the general algorithmic scheme of the **SiReNe** software.

In addition, two variants were implemented, with or without storage of the neurons connectivity. The most efficient combination of time-stepping approach and connectivity management, according to execution time as well as memory consumption, is the event-driven (spike-driven) approach with connectivity generation.

Finally, parallel multi-threading has been added via the OpenMP API. The parallel strategy mainly consists in distributing the computations related to the neurons over the threads. In particular, parallelism is exploited in the computation of the derivatives, the spike detections and the synaptic current updates. For the parallel generation of neuron connections, it is mandatory to use a thread-safe random generator. Thus, we have added the required software layer over the random generator described in section 3.2.1.

### 3.6. Experimental Context

The simulations presented below have been done on a Dell R720 server under Linux Debian 4.9 amd64 with 2 Intel(R) Xeon(R) CPU E5-2640 v2 @ 2.00 GHz with 8 cores each, and 128GB RAM. Times are measured with the OpenMP function omp_get_wtime() and the program is compiled with gcc 6.3.0 and the -O3 optimization level. All simulations presented in this section are done in sequential (one thread) save for the ones related to the performance evaluation of the parallel version (multiple threads).

## 4. Results

### 4.1. Order of the Method

The order of the simulation method is estimated on the last spike of a 20 ms simulation fired by a single neuron over repeated trials (*N*_*t*_ = 10) with random initialization. We simulate different time-steps between Δ*t* = 5*e*^−5^ ms and Δ*t* = 0.01 ms. Three different methods are evaluated. In addition to the lines intersection (**RK2Lines**) and the maximum of Bézier curve (**RK2Bezier**) described in section 3.3, we add the simplest method in which the spike is aligned to the time-step (**RK2Threshold**) when the threshold is exceeded. A complete list of the simulation parameters related to this experiment (named *Method order*) is given in Supplementary Material.

Although the RK2 numerical scheme is used, the simulation order is not systematically two, as it depends on the spike detection method (Hansel et al., 1998). As there is no analytical solution to calculate the error of our model, we use as reference a simulation with a very small time-step Δ*t* = 1*e*^−7^ ms. Indeed, for such small time-step, the error can be considered negligible. Thereby, in this context the error over the spike time (ϵ_*t*_) is defined as

(11) $$\begin{array}{l} \epsilon_{t}=\frac{1}{N_{t}}\overset{N_{t}}{\underset{i=0}{\sum }}|{\hat{t}}_{i}-t_{i}| \end{array}$$

where *N*_*t*_ is the number of trials, ${\hat{t}}_{i}$ is the time of the last spike of the neuron within the simulated period for a given Δ*t*, and *t*_*i*_ is the corresponding time for the reference simulation with Δ*t* = 1*e*^−7^ ms.

**Figure 5:**
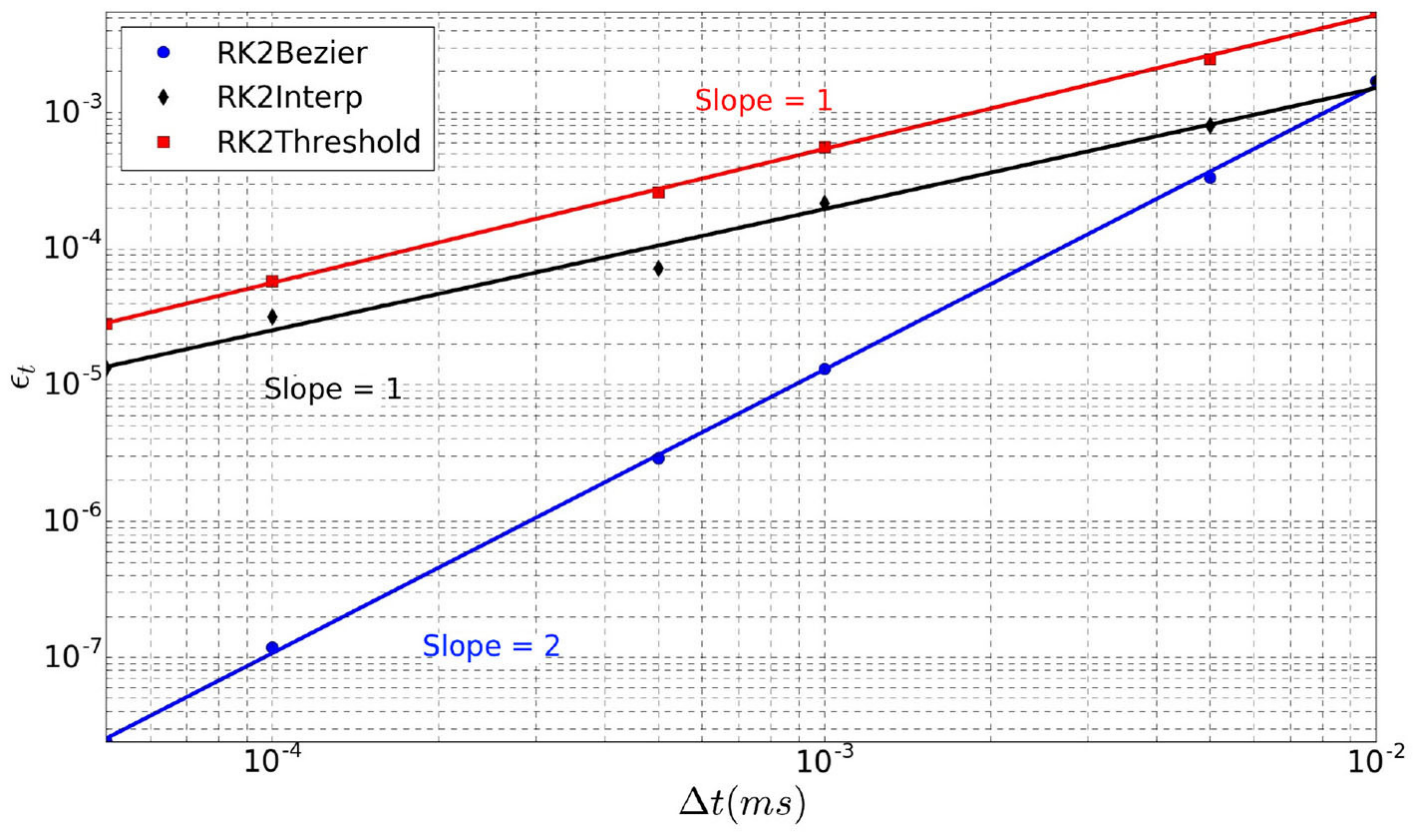
Error ϵ_*t*_ over spike time in function of time-step Δ*t*, for the three spike detection methods.

The error ϵ_*t*_ over the spike time is depicted in Figure 5 for the three interpolation methods as a function of the time-step Δ*t* (*log* scales). For each method, linear regression provides a line whose slope represents its order. Indeed, **RK2Threshold** and **RK2Lines** are first order methods (error is O(Δt)). The **RK2Bezier** detection method is more accurate and preserves the order two of the original RK2 integration method (error is $O\left(\Delta t^{2}\right)$).

### 4.2. Performance Comparison of the Different Methods

As described before, two distinct simulation approaches are available in **SiReNe**: the classical time-stepping approach and the event-driven updating one. In addition, it is possible either to store the connectivity or to generate it on-demand. When combining those two aspects, we obtain four algorithmic variants. In order to determine which of the four approaches is the most efficient, we compare them in terms of memory consumption and execution time. Also, in order to compare our simulator to a reference from the community, we add simulations obtained with **BRIAN2**, which is one of the most used simulator for spiking neurons (Goodman and Brette, 2008).

**Figure 6:**
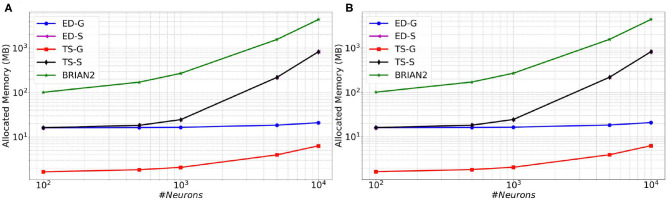
Memory consumption as a function of the size of a MSN network with 100% of connectivity **(A)** or with 30% connectivity **(B)** during 100 ms biological simulation, for the TS-S, TS-G, ED-S, and ED-G simulation approaches and the **BRIAN2** simulator.

**Figure 7:**
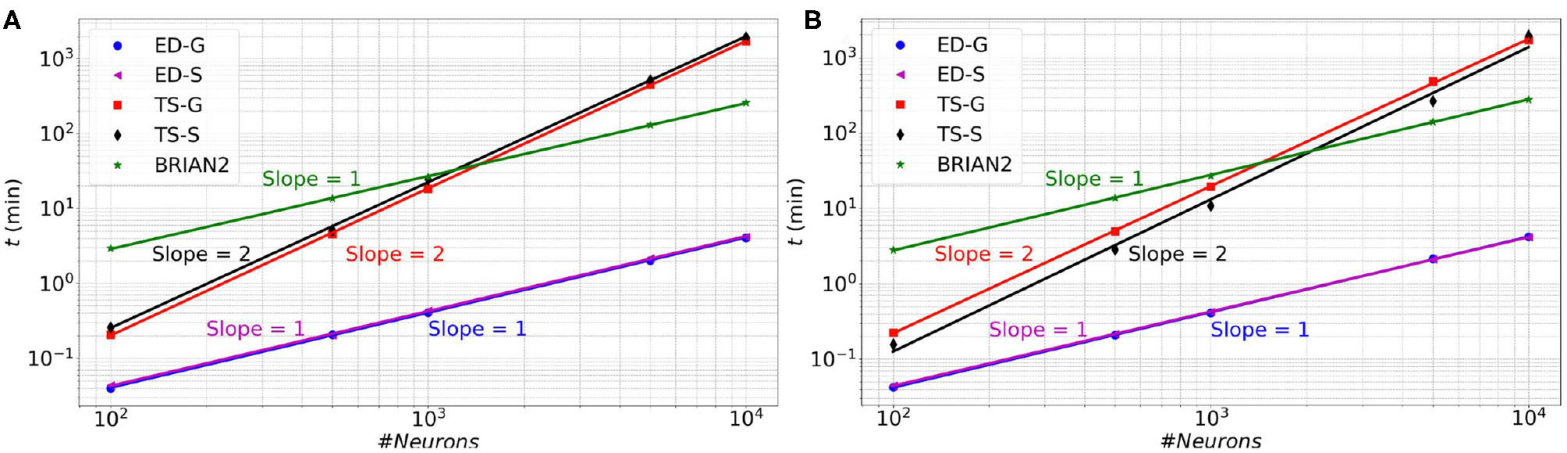
Execution time as a function of the size of a MSN network with 100% of connectivity **(A)** or with 30% connectivity **(B)** during 100 ms biological simulation, for the TS-S, TS-G, ED-S, and ED-G simulation approaches and the **BRIAN2** simulator.

In Figure 6 (resp. Figure 7), memory consumption (resp. execution time), are given as a function of the size of a MSN network with 100% of connectivity (A) or 30% of connectivity (B) during 100 ms biological time. The five compared approaches are the classical **T**ime-**S**tepping approach with connectivity **S**torage (TS-S), the same approach with connectivity **G**eneration (TS-G), the **E**vent-**D**riven updating with connectivity **S**torage (ED-S), the same approach with connectivity **G**eneration (ED-G), and the approach used in the **BRIAN2** simulator. A complete list of the simulation parameters related to this experiment (named *Memory and time*) is given in Supplementary Material.

From these results, it appears that storing the connectivity is definitely inappropriate, as it sharply increases the memory consumption (Figure 6). As expected, the method with storage has a memory consumption that scales in a polynomial way with the number of neurons while the scaling is linear for the method without storage.

Concerning the performance, we observe a relationship between the execution time and the number of neurons. The time varies as a power of the number of neurons that is *t*∝*n*^*k*^, where *t* is the execution time, *n* the number of neurons and *k* the exponent of the power law. In Figure 7, the slopes of the **ED-S**, **ED-G** methods and **BRIAN2** are of order one (*k* = 1), whereas for the other two methods the slopes are of order two (*k* = 2). It means that the execution time increases linearly for **ED-S**, **ED-G**, and **BRIAN2** and quadratically for the two other methods. Nevertheless, **BRIAN2** has a significantly larger simulation time compared to the **ED**-methods. The **ED-S** and **ED-G** methods have similar simulation times. However, for full connectivity (Figure 7A), we observe a slight gain in execution time with the non-storing version that comes from the possibility to avoid the on-demand generation in that particular case. When the connection probability is less than 1 (Figure 7B), the connectivity generation induces a slight overhead on the execution time. However, we observe that this overhead tends to decrease when the number of neurons increases.

For a last comparison between **SiReNe** and **Brian**, we implemented in **SiReNe** the COBAHH network model described in article (Brette, 2007). The COBAHH model is a network of 4,000 excitatory-inhibitory neurons (80% excitatory and 20% inhibitory Hodgkin-Huxley-type neurons with full connectivity). In Brette (2007), this benchmark model was simulated in **Brian** with Euler integration (0.01 ms step-size) and the spikes were detected by threshold crossing at –20 mV with 3 ms refractory period and alignment of the crossing events on the step-size. Although this detection method associated with Euler integration leads to a precision of order one, we implemented them in **SiReNe** for a fair comparison with **Brian**. We simulated this benchmark model for 1 s of biological time on the same machine (Dell R720 server, section 3.6) for both **Brian** and **SiReNe**. The simulation time is more or less the same, ~1 min. The memory consumption was 18 MB for **SiReNe** vs. 375 MB for **BRIAN**.

### 4.3. Parallel Computing Performance

In order to evaluate the performance of the parallel multi-threaded version of **SiReNe**, the memory consumption and execution times are measured as a function of the number of threads for the best approach (event-driven). The test case is the simulation of 10000 MSN neurons with 504 incoming connections each, during a biological time of 1 s. A complete list of the simulation parameters related to this experiment (named *Parallel performance*) is given in Supplementary Material.

**Figure 8:**
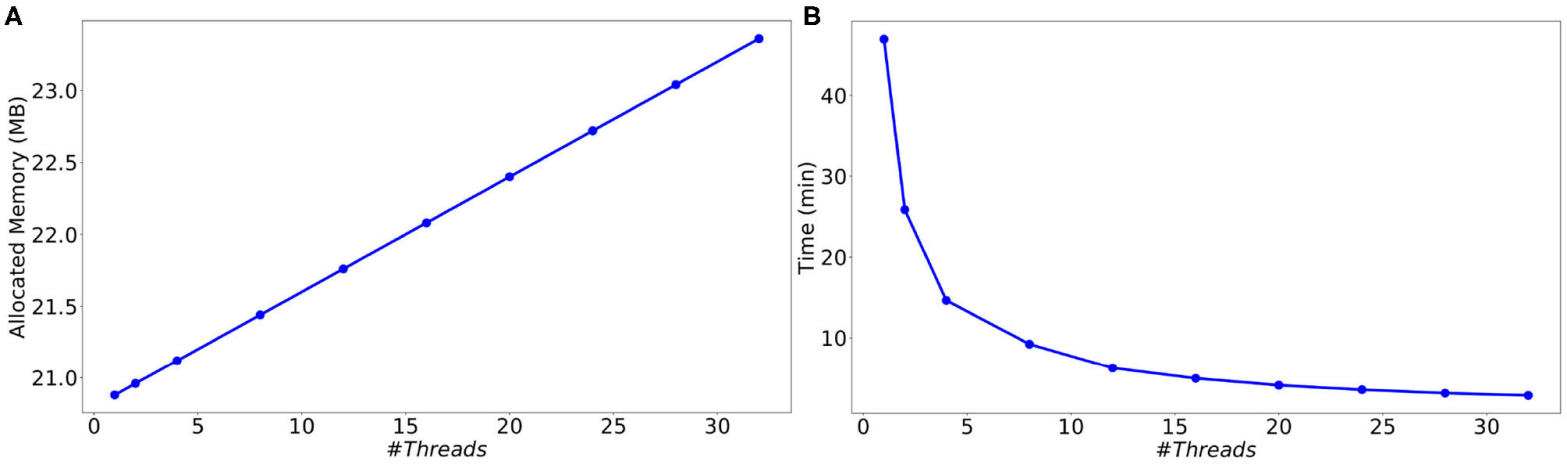
Memory consumption **(A)** and execution time **(B)** as a function of the number of threads for the simulation of a MSN network with 10 k neurons (504 incoming connections per neuron) during 1 s biological time.

**Figure 9:**
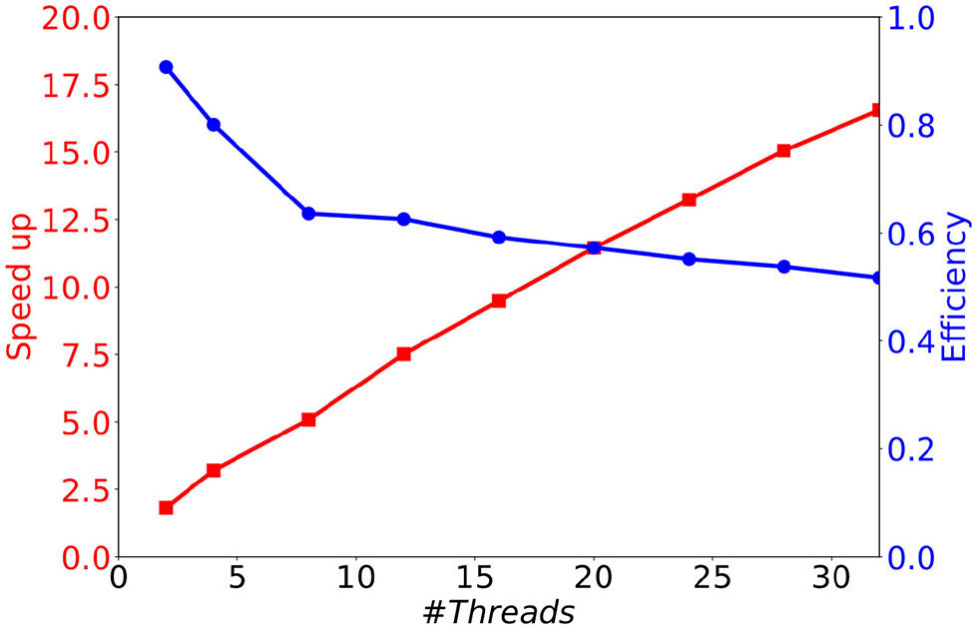
Speedup and efficiency of a 1 s biological time simulation of 10 k MSN neurons with 504 incoming connections, as a function of the number of threads.

As can be seen in Figure 8A, although the memory consumption increases slightly with the number of threads, it stays very limited. Indeed, the additional consumption with 32 threads compared to 1 thread is smaller than 3 Mb (less than 12 % of the initial consumption). This comes from the fact that only the conductance factors between neurons (see Equation 4) are duplicated to support their concurrent updates by the threads. Concerning the execution time, we observe in Figure 8B a significant decrease when the number of threads increases. This result shows that the parallel version provides a significant gain of time. However, the time decrease is less important than expected, leading to moderate speed-ups for large numbers of threads (≥16), and a faster decrease of the parallel efficiency (speedup over the number of threads) than expected (see Figure 9). This is due to the irregular inter-dependencies between neurons, that induce irregular memory access patterns, penalizing the parallel accesses. This issue deserves a complete detailed study that is planed as a future work.

### 4.4. Simulation of the Striatum Model

**Figure 10:**
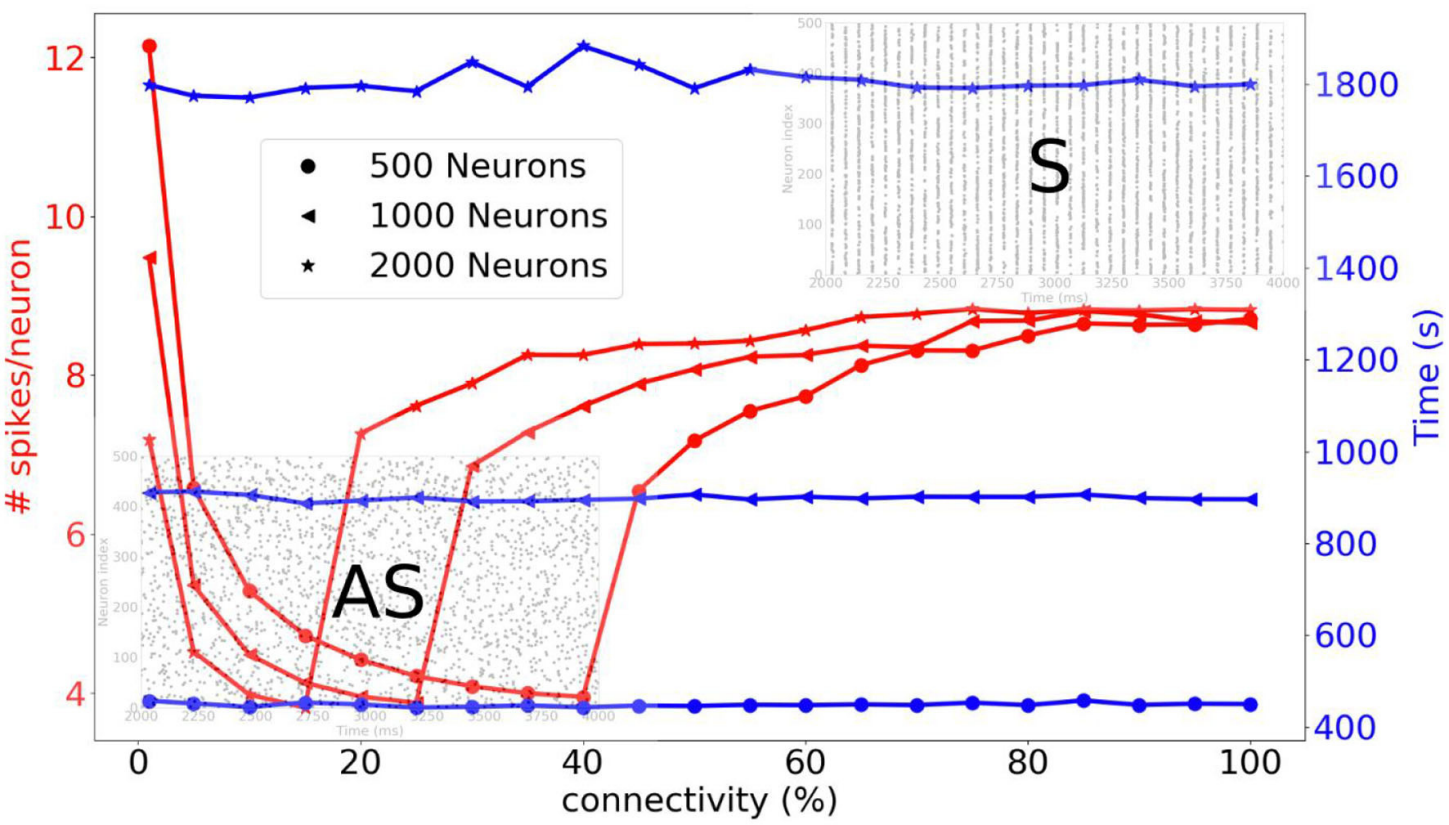
Three different neural networks are simulated, i.e., 500, 1,000, and 2,000 neurons. The average number of spikes per neuron and simulation time are compared as function of the connectivity. For a sparse connectivity, we observe an asynchronous regime [raster plot indicated with (**AS**)]. For dense connectivity, we observe synchronous regime [raster plot indicated with (**S**)]. An abrupt transition occurs at a certain percentage of connectivity.

Before going on to the simulation of the striatum, we simulate three different sizes of neural networks (500, 1,000, and 2,000 neurons) and we compare the average number of spikes per neuron and the simulation time as a function of connectivity. A complete list of the simulation parameters related to this experiment (named *Network comparison*) is given in Supplementary Material. In Figure 10, we can see that for a sparse connectivity, the neural network has an asynchronous regime and for a dense connectivity, the neural network has a synchronous regime. For the respective neural network sizes, abrupt transitions of the number of spikes can be observed at different percentages of connectivity. The simulation times are quite stable when the connectivity varies.

For the comparison of the results with (McCarthy et al., 2011), two types of plots are produced: the raster plot of the spiking neurons and the power spectral density of the LFP model. The former displays the set of spiking neurons at each time step. The latter draws the LFP power as a function of the spiking frequency.

The test case is a MSN network with 1.3 million neurons, simulated during 4 s biological time, either under normal or Parkinson conditions. The simulation time is 10 h per second of biological time with a time-step of 0.01 ms. The memory allocation is less than 1 GB. The normal conditions correspond to an applied input *I*_*app*_ = 1.19 μA and a maximal M-current conductance ḡ_*M*_ = 1.34 ms. To obtain Parkinson conditions, ḡ_*M*_ is decreased to 1.1 ms. A complete list of the simulation parameters related to this experiment (named *Reference (striatum)*) is given in Supplementary Material.

**Figure 11:**
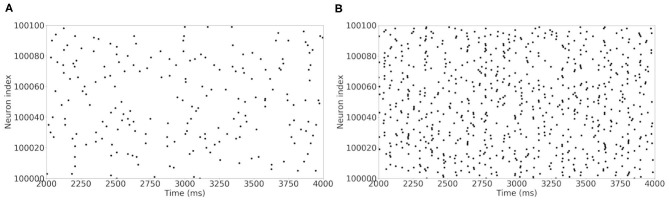
Raster plot of the large MSN test case for neurons in interval [100,000 : 100,100]. Spiking neurons at each time-steps under normal conditions **(A)** and Parkinson's conditions **(B)** for the simulation of a MSN network at scale 1 of the rat, during 4 s biological time.

**Figure 12:**
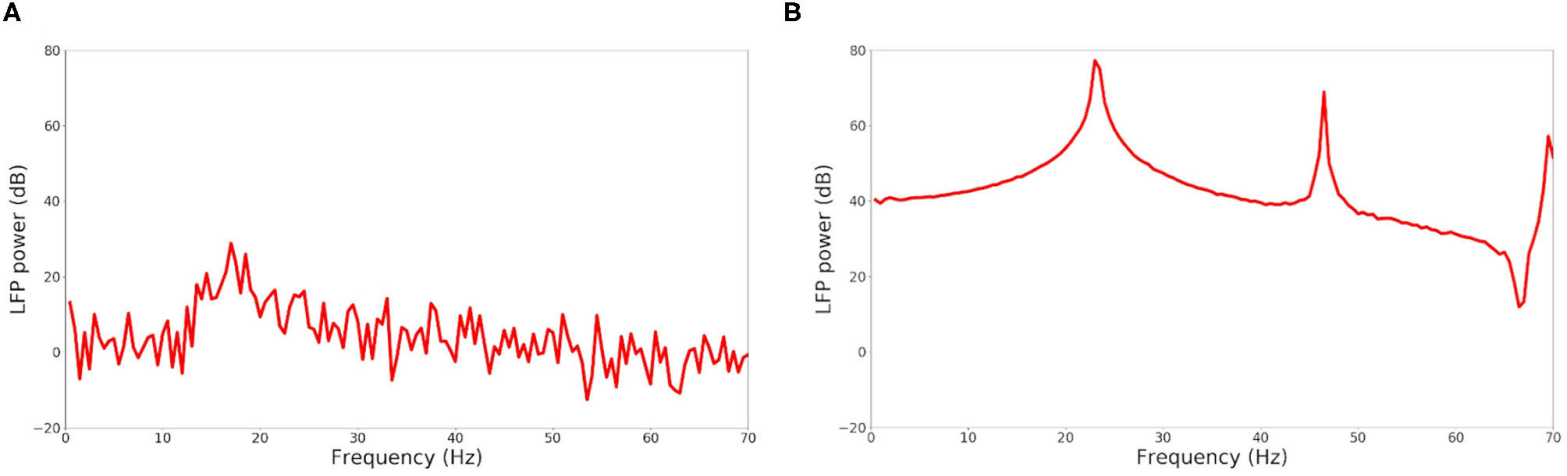
LFP power in function of the frequency under normal conditions **(A)** and Parkinson's conditions **(B)** for the simulation of a MSN network at scale 1 of the rat, during 4 s biological time.

The raster plot in Figure 11A shows a MSN simulation under normal conditions. The average spiking rate for the neurons is 0.94 ± 0.63 Hz, which is coherent with the average MSN spiking rate *in vivo* (1.1 ± 0.18 Hz, Kish et al., 1999 and 0.96 ± 0.03 Hz, McCarthy et al., 2011). Figure 11B shows the simulation of a parkinsonian striatum. As expected, there is a pathological synchronization of the neurons, which is representative of Parkinson's disease. The average spiking rate raises up to 3.71 ± 1.03 Hz (2.11 ± 0.43 Hz in Kish et al., 1999 and 4.9 ± 0.15 Hz in McCarthy et al., 2011). The mean firing rates are significantly different between healthy and Parkinson conditions (*p* < 1*e* − 3, *t*-test). Concerning the LFP spectrum, it can be seen that we obtain similar qualitative behaviors than those reported in McCarthy et al. (2011) under normal and parkinsonian conditions. In Figure 12A (normal conditions), a small peak can be observed (max of 30 dB at 17 Hz), revealing a weak synchronized state. In Figure 12B (Parkinson's conditions), the LFP reaches higher β band oscillations (≈80 dB at 23 Hz), which is representative of Parkinson's pathological oscillations with strong neural synchronization (McCarthy et al., 2011).

The similar behavior of our simulations to those of McCarthy et al. (2011) (increase in MSN spiking frequency and LFP β power in Parkinson's state) validates the event-driven updating version of the **SiReNe** software to simulate large-scale networks (>10^6^ neurons in our case vs. 100 neurons for McCarthy et al., 2011). It has to be mentioned that no comparison could be done with **Brian2** for such a large network, as it exceeds the memory capacity of our server (128 GB RAM) to store the connectivity.

## 5. Conclusion

An event-driven updating approach for the simulation of neural networks has been presented. It mainly consists in updating post-synaptic currents only for spiking neurons, thus reducing the computational cost. Moreover, the addition of a pseudo-random generation of the neurons connectivity allows for a significant reduction of memory cost, passing from *O* (*n*^2^) to *O* (*n*) complexity, for *n* neurons in the system. Finally, two interpolation methods (linear intersection and Bézier curve) have been compared for the computation of spike times.

A set of experiments has been conducted to evaluate the validity of the approach as well as its accuracy and performance. The validity is confirmed by a comparison to the reference work of McCarthy et al. (2011) modeling a basal ganglia MSN network in healthy and Parkinsonian conditions. Concerning the accuracy, we conclude that to preserve the order 2 of the Runge-Kutta method, it is mandatory to use an order 2 interpolation method of the spike times. In our case, we have opted for quadratic Bézier curves as they are controlled by the tangents at end points and they have limited computational cost. Finally, the performance study is 2-fold. It shows that the event-driven updating approach is not only faster than the classical time-stepping one, but that it has a better performance scaling according to the number of neurons. Also, the connectivity generation seems to have a limited impact on the performance, especially when the number of neurons increases.

One limitation in the event-driven connectivity generation concerns the inclusion of plasticity rules, such as STDP (Spike-Time Dependent Plasticity). Indeed, such rules are difficult to implement in an event-driven strategy because the synaptic conductances have to be stored in order to be updated at each spike time, which would cause a large increase in memory consumption. However, in many neuroscience studies, the plasticity is not a crucial key-point as a snapshot of a neural network is simulated over a short period of time in order to analyze its functional behavior rather than its evolution. This is true in the simulations of our Striatum network, as pathological oscillations in the Parkinson state are not obtained from synaptic plasticity but by changing the intrinsic M-channel conductance of the MSN neurons (McCarthy et al., 2011). Other limitations of the current version of **SiReNe**, like the event-driven generation of distance-dependent connectivity or synaptic delays, are less difficult to tackle and should be addressed with a limited impact over performance.

Another contribution concerns the overall numerical methods used to simulate Hodgkin-Huxley based neuron models. This type of neurons is sensitive to the time-step value because with large time-steps (>10^−2^ ms), the significance of the derivatives used to deduce next step values are much weaker than with small time-steps (10^−7^ ms) (Moore and Ramon, 1974). In fact, when there is no sharp variation of the membrane potential inside a time-step, the derivative at the beginning is close to the average value of derivatives inside the step, leading to a good approximation at the end of the step. However, when the potential sharply varies, typically when the neuron is spiking, the derivative at the beginning of the step is much lower than the average value of the derivatives inside the step, leading to an underestimation of the potential at the end of the step. In order to stretch the limits induced by this problem, a specific process is under consideration for inclusion in the **SiReNe** software. Although a complete study is necessary to fully evaluate the gain and interest of such corrective process, preliminary experiments show promising results. A future work will be dedicated to this subject.

In this paper, the main features of the event-driven updating and connectivity generation have been studied. The combination of this approach with time-stepping numerical integration of the Hodgkin-Huxley equations forms a very pertinent solution to efficiently and accurately simulate large neural networks with limited computing resources (single server). However, there is still room for improvements and extensions. As a few examples, the reduction of irregular memory accesses in the multi-threaded version, extensions of the parallel version to support multiple machines and/or GPUs, the inclusion of distance-dependent connectivity, synaptic delays as well as the corrective process of the numerical method when using large time-steps would deserve further studies. All those aspects will be considered as future works.

## Data Availability Statement

The datasets generated for this study are available on request to the corresponding author.

## Author Contributions

NA, SC-V, LB, and DM contributed to the conception of the simulator. NA, SC-V, and DM contributed to the development of the Sirene software. NA conducted the experiments to validate the models. All authors contributed to manuscript revision, read, and approved the submitted version.

## Conflict of Interest

The authors declare that the research was conducted in the absence of any commercial or financial relationships that could be construed as a potential conflict of interest.
