# Supplementary material for: Simulation of Large Scale Neural Models With Event-Driven Connectivity Generation
Source: Front Neuroinform. 2020 Oct 14;14:522000. doi: 10.3389/fninf.2020.522000 (PMC7591773; doi:10.3389/fninf.2020.522000)
Supplement: Supplementary file 1 [file Table_1.pdf]

Supplementary Material

In order to provide reproducible results, the relevant cell and synaptic parameters are given for each simulation (Tables S1, S2, S3 and S4). Each experiment mentioned in the paper is given a name and the list of its related figures:

- *Method order*: comparison of the simulation accuracy orders between different spike detection methods (Figure 5).
- *Memory and time*: comparison of memory consumption and execution times between **BRIAN2** and the event driven updating and the time-stepping approaches with and without connectivity storage, proposed in **SiReNe** (Figures 6 and 7).
- *Parallel performance*: performance evaluation of the parallel version of **SiReNe** (Figures 8 and 9).
- *Network comparison*: comparison of the average number of spikes per neuron and the simulation times as a function of connectivity, for three different network sizes (Figure 10).
- *Reference (striatum)*: simulation of the MSN network with 1.3M neurons (Figures 11 and 12).

Table S1 provides the general parameters for each simulation. The first line gives the corresponding figures in Section 4. The other lines correspond to the different simulation parameters, like the interpolation method used to compute the spike times, the numerical method, the membrane potential threshold for the spike detection, the simulation start and duration, the time-step and the number of threads used during the simulation.

	Experimental contexts				
General Parameters	**Reference (striatum)**	**Method order**	**Memory and time**	**Parallel performance**	**Network comparison**
Figure(s)	11 & 12	5	6 & 7	8 & 9	10
Network size	1.3M	1	$100 - 10k$	10k	$100 - 2000$
Interpolation type	Bézier curve	Lines Intersection/ Threshold	Bézier curve	Bézier curve	Bézier curve
Numerical method	RK2	RK2	RK2	RK2	RK2
Spike threshold [mV]	0	0	0	0	0
Duration [ms]	4000	20	100	1000	4000
time-step [ms]	0.01	$0.01 - 5e^{-5}$	0.005	0.005	0.005
#threads	32	1	1	$1 - 32$	1

Table S1. General parameters of the presented experiments.

In Table S2 are given the input current parameters. The input current types are either a constant current or a step current with noise. For the step current, a negative current is introduced in the first seconds. Then, a positive current, smaller than one, is injected. In addition, a noise is applied over the step time in order to avoid a similar step for all the neurons. Finally, a noise amplitude is also added to the input current.

	Experimental contexts				
Input current Parameters	**Reference (striatum)**	**Method order**	**Memory and time**	**Parallel performance**	**Network comparison**
Input Current type	Noisy step	Constant	Noisy step	Noisy step	Noisy step
Step time [ms]	500	/	50	100	100
Noise	$\times[0, 1]$	/	$\times[0, 1]$	$\times[0, 1]$	$\times[0, 1]$
I_{input} $[\mu A]$	/	0.5	/	/	/
I_{input} $[\mu A]$ *the* 1st *ms*	-10	/	-10	-10	-10
I_{input} $[\mu A]$ *the* 2nd *ms*	1.19	/	0.5	0.4	0.5
Noise amplitude [%]	300	/	300	300	300

Table S2. Input Current parameters of the presented experiments.

As shown in Table S3, the neuron initialization values and parameters are the same for all the experiments. A heterogeneity percentage is added to the initialization parameters. The only parameter that varies from one experiment to another is the maximal conductance g_m. For *Memory and time* and *Parallel performance*, the conductance is 0.25 mS, while it is 0.6 mS for *Network comparison*.

Neuron Initialization Parameters	**For all experiments**	***Neuron Parameters***	**For all experiments**
V $[mV]$	-63.826	C_m $[pF]$	1.0
m	0.027781	g_L $[mS]$	0.1
h	0.990915	E_L $[mV]$	-67.0
n	0.062045	g_{Na} $[mS]$	100.0
p	0.022788	E_{Na} $[mV]$	50.0
Heterogeneity [%]	10	g_K $[mS]$	80.0
		E_K $[mV]$	-100.0
		g_M $[mS]$ *(normal)*	1.34
		g_M $[mS]$ *(Parkinson)*	1.1

Table S3. Neurons initialization parameters and neurons parameters for all the presented experiments.

The synaptic parameters are provided in Table S4. The synaptic conductance is divided by the number of connections. The **BRIAN2** parameters are not provided as they are the same as the *Memory and time* experiment. The only difference lies in the spike time computation, since the only available approach in **BRIAN2** is the alignment to the time-step.

Synaptic Parameters	**Experimental contexts** Reference (striatum)	Method order	Memory and time	Parallel performance	Network comparison
Network size	1.3M	1	$100 - 10$k	10k	$100 - 2000$
connections [%]	0.0004	/	$30 - 100$	0.05	$1 - 100$
τ $[ms]$	15	/	12	12	12
E_{syn} $[mV]$	-80.0	/	-80.0	-80.0	-80.0
g_{syn} $[nS]/\#connections$	$0.1 - 0.6$	/	0.6	0.25	$0.2 - 1.0$

Table S4. Synaptic parameters of the presented experiments.
